# A qualitative study into the use of formal services for dementia by carers from culturally and linguistically diverse (CALD) communities

**DOI:** 10.1186/1472-6963-12-354

**Published:** 2012-10-09

**Authors:** Christopher Shanley, Desiree Boughtwood, Jon Adams, Yvonne Santalucia, Helena Kyriazopoulos, Dimity Pond, Jeffrey Rowland

**Affiliations:** 1 Liverpool Hospital, University of New South Wales, Elizabeth St, Liverpool, 2170, Australia; 2 Cumberland Prospect Multicultural Health Project, Western Sydney Local Health District, Fleet St, Parramatta, 2150, Australia; 3 Faculty of Health, University of Technology Sydney, Jones Street, Ultimo, 2007, Australia; 4 Multicultural Health Service, South Western Sydney Local Health District, Elizabeth St, Liverpool, 2170, Australia; 5 Alzheimer’s Australia South Australia, Conyngham Street, Glenside, 5065, Australia; 6 Faculty of Health, University of Newcastle, University Drive, Callaghan, 2308, Australia; 7 Department of Internal Medicine, Prince Charles Hospital, Rode Road, Chermside, 4032, Australia

**Keywords:** Dementia, Ethnic minority, CALD, Carers, Services, Australia

## Abstract

**Background:**

People with dementia and their family carers need to be able to access formal services in the community to help maintain their wellbeing and independence. While knowing about and navigating one’s way through service systems is difficult for most people, it is particularly difficult for people from culturally and linguistically diverse (CALD) communities. This study addresses a lack of literature on the use of formal services for dementia by people from CALD backgrounds by examining the experiences and perceptions of dementia caregiving within four CALD communities – Italian, Chinese, Spanish and Arabic-speaking – in south western Sydney, Australia.

**Methods:**

The study used a qualitative design and the methods included focus groups with family carers and one-to-one interviews with bilingual/bicultural community workers, bilingual general practitioners and geriatricians. A total of 121 family carers participated in 15 focus groups and interviews were held with 60 health professionals. All fieldwork was audiotaped, transcribed and subjected to thematic analysis.

**Results:**

People from CALD communities are often unfamiliar with the concept of formal services and there may be strong cultural norms about maintaining care within the family, rather than relying on external services. CALD communities often have limited knowledge of services. There is a preference for services that will allow families to keep their relative at home, for safety as well as cultural reasons, and they are particularly reluctant to use residential care. While there is a preference for ethno-specific or multicultural services, mainstream services also need to ensure they are more flexible in providing culturally appropriate care. Positive outcomes occur when ethno-specific services work in partnership with mainstream programs. Dementia service providers need to develop a trusting relationship with their local CALD communities and promote their services in a way that is understandable and culturally acceptable to members of these communities.

**Conclusions:**

While members of CALD communities may have difficulties accessing formal services, they will use them if they are culturally and linguistically appropriate and can meet their needs. There are a number of ways to improve service provision to CALD communities and the responsibility for this needs to be shared by a range of stakeholders.

## Background

For people with dementia and their family carers it is essential that they know about, and are able to access, the range of dementia-related services available to them. This is often particularly challenging for people from culturally and linguistically diverse (CALD) communities who may lack information about such services or may not perceive the relevance of the services to their situation [1]. Australia is a highly multicultural society and the number of older people from CALD communities with dementia will continue to increase [2]. It is therefore essential that policy makers and service providers have as clear an understanding as possible of the issues confronting people from CALD communities so that dementia-related services are relevant and accessible to people from these communities.

Much of the research into the dementia-related service needs and service provision within multicultural communities has been undertaken in the US or United Kingdom [3]. Some of the key findings from this work are that: differences in language and cultural background of the person living with dementia (PLWD) and the clinician conducting the dementia assessment may impact on accuracy of the diagnosis [4]; CALD communities may have different understandings of dementia than the Western medical understanding and this may affect their approach to services [5]; stigma, lack of knowledge about and negative experiences of services and language barriers all contribute to a low use of services [6,7].

Mukadam, Cooper and Livingston [8] reviewed 13 papers on how and why people from CALD communities present to specialist dementia services. They concluded that people often present to dementia services later in the condition and that there are a number of barriers that people face. These include: not conceptualising dementia as an illness; believing dementia was a normal consequence of ageing; thinking dementia had spiritual, psychological, physical health or social causes; feeling that caring for the person with dementia was a personal or family responsibility; experiences of shame and stigma within the community; believing there was nothing that could be done to help; and negative experiences of healthcare services. Recognition of dementia as an illness and knowledge about dementia facilitated accessing help.

Cooper et al [9] undertook a systematic review and meta-analysis of ethnic differences in the use of dementia treatment, care and research. They included 33 papers in their review. They concluded that – compared to the general population – people from CALD communities accessed diagnostic services later in their illness, and once they did receive a diagnosis, were less likely to access anti-dementia medication, research trials and 24-hour care.

Although some of the issues identified by US and UK researchers may apply to CALD communities in Australia, similarities should not be assumed, both because of the make-up of the communities and differences between health systems in the different countries (Barker, personal communication, 2010). Australian research findings include that: CALD communities have lower use of services [10-13]; and CALD people living with dementia present with more advanced symptoms and in poorer health than Anglo elderly [12-14].

One body of Australian literature, focusing on residential aged care, examined the relative roles of ethno-specific (services catering for a specific CALD group) and mainstream services. Westbrook and Legge [15] found that both CALD residents and family caregivers preferred ethno-specific services, because of the food provided and because the facilities encouraged family members to be involved in care.

Studies have shown more positive outcomes for CALD residents of ethno-specific facilities, compared to those in mainstream facilities. Runci, Redman and O’Connor [16] identified that residents in ethno-specific homes were prescribed less benzodiazepines and engaged in more social interaction than residents of mainstream homes. Goh, Low and Brodaty [17] compared levels of depression amongst Chinese residents in ethno-specific and mainstream facilities. They found that while the rates of depression were similar, the drug treatments used to treat depression were more appropriate and effective in the ethno-specific settings.

The potential for mainstream facilities to provide culturally appropriate care has been outlined [18] and other authors have argued that there is a need for both ethno-specific and mainstream facilities as well as a partnership model where both types of facilities support each other and work together for the benefit of clients and families [19].

Australian studies examining the perceptions and use of dementia services can also be found in the grey literature (written reports that may not be publically available or published in academic peer-reviewed formats). This literature suggests that CALD communities underutilise services in comparison to Anglo populations [20], due to a lack of knowledge of dementia as a disease, and families being unaware or unwilling to use services [21]. Some CALD communities are willing to use services such as community nursing and culturally specific respite, but not residential aged care [21,22].

While there has been a certain amount of research undertaken – as outlined above – further research on the use of dementia-related services amongst CALD communities has been put forward as a priority research area [23]. This is particularly the case in view of the projected increase in people with dementia from CALD communities over the next forty years [2].

In response to this identified need, we undertook a study of dementia caregiving in the Arabic, Chinese, Italian and Spanish-speaking communities living in south western Sydney, Australia. These communities were chosen because they are some of the larger groups in the local area and are distinct from each other in terms of language, migration history and acculturation. One broad aim of the study – and the focus of this paper – was to explore the perceptions and experiences of CALD family carers and a range of health professionals with regards to the use of dementia-related formal services. The research questions this paper aims to address are:

1) What dementia-related services do people from CALD communities use?

2) What issues do people from CALD communities face in using dementia-related services?

3) How can the provision of dementia-related services to CALD communities be improved?

Data was gathered from family carers, bi-lingual general practitioners, geriatricians and bilingual/bicultural workers providing a health or welfare service to one language community or CALD communities generally. The range of dementia-related services covered in this project included in-home personal care, in-home domestic help, in-home and centre-based respite, and residential care. Ethno-specific services provide care to one cultural or language community; multicultural services provide care to several different CALD communities; and mainstream services cater to the whole broader community.

## Methods

### Research questions and design

The project employed a qualitative design focused upon examining the perceptions and experiences of family carers and a range of relevant practitioners to build an understanding of issues related to the use of formal services for people with dementia [24]. Such an approach is well suited to exploring areas where little previous research exists, as is the case in this project.

### Ethics, participants and recruitment

The project received approval from the University of Queensland Human Research Ethics Committee and the Sydney South West Area Health Service (Western Zone) Human Research Ethics Committee.

There were two broad categories of participants in the project. The first of these were family carers, who were accessed by focus groups. The second was three groups of health professionals – bilingual/bicultural workers, bilingual general practitioners and geriatricians – who were accessed by one-to-one interviews.

In terms of the family carers, bilingual fieldworkers were the key persons responsible for recruitment and facilitation of the focus groups. One fieldworker was employed for each of the four communities. They all had undergraduate qualifications and extensive experience in education and community support roles within their communities. The fieldworkers received training and support from the research team in facilitation of the focus groups. While the focus groups were facilitated by the bilingual fieldworkers, a member of the research team (DB) was an observer in all groups. DB sat at the back of the group and was accompanied by an interpreter working just with her who quietly explained what was being discussed within the group. This provided an opportunity to maintain ongoing training and support and ensure the quality of the fieldworkers’ tasks throughout the research process.

Family carers were eligible to participate in the study if they had an immediate or extended family member with dementia whom they cared for within the last 12 months. The person with dementia needed to speak a language other than English at home. Flyers advertising the project and calling for participants were translated into the four languages. The bilingual fieldworkers invested considerable effort in liaising directly with CALD community organizations to promote the project. Other means of recruitment included church newsletters, stories and advertisements in ethnic media, and liaison with local bilingual medical practitioners.

The focus groups utilized an interview guide that included questions on issues such the community understandings of dementia, access to dementia-related information, how carers dealt with initial signs of dementia through to diagnosis, use of dementia services and what forms of support carers knew about and used. While this interview guide was used to provide a standard approach across the focus groups, the facilitators allowed considerable flexibility in discussion to allow new issues to be raised by participants.

A total of 121 family carers participated in fifteen focus groups. Of these, 19 were Arabic, 37 Chinese, 40 Italian and 25 Spanish-speaking. Eighty-eight carers were women and 33 men. The youngest carer was 17 and the oldest was 90 years old. Twelve of the groups were conducted in the community language and three in English, at the request of participants. While the groups all used the same schedule of questions, the cultural backgrounds of participants were respected through using a facilitator familiar with cultural norms of communication, venues familiar to the community and culturally appropriate refreshments.

In terms of the interviews with health professionals, all these were undertaken by one member of the research team (DB). The three professional groups were included because they were seen as playing a central role in care of people with dementia. Recruitment was based on lists of professionals within the geographical area of the study. In the case of bilingual/bicultural workers, recruitment aimed to get a mixture of participants from the four language communities and different service types. For the bilingual GPs, a list was drawn up from local networks and GPs from each community approached directly. For geriatricians, the participants represented almost all the geriatricians within the geographical area.

An interview guide was generated through identifying gaps in the published and gray literature as well as consultations with community leaders in each of the four communities studied. Some of the questions sought the professionals’ perspectives on questions asked of carers in the focus groups, such as understandings of dementia, access to information and use of services. Other questions focused on the health professionals’ role in providing dementia education and support to CALD communities.

Bilingual/bicultural worker is a generic term to cover a number of positions in the health/welfare sector. These workers provide culturally and linguistically appropriate services to their communities. Their roles include health education and promotion, community development, running information and support groups and, to a more limited extent, casework and counselling. Twenty-four bilingual/bicultural workers participated; six were Arabic, seven Chinese, seven Spanish and four Italian. Two were male and the remainder female. Three participants were employed in the state health service; two were in multicultural and five in mainstream organizations; 12 in ethno-specific community services; and two in residential ethno-specific services. The length of time employed as a bilingual/bicultural worker ranged from four to twenty-five years.

Sixteen bilingual GPs were interviewed. Four were Arabic, three Chinese, four Italian and five Spanish-speaking. One was a sole practitioner, 10 worked in language specific practices and five in generalist medical centres. 12 were male and four female. Their length of practice ranged from 13 to 37 years.

Twenty geriatricians participated in interviews. All were employed in hospitals and two also worked in private practice. Although not selected on this basis, two geriatricians spoke Arabic, two Chinese languages and one spoke Spanish. There was significant variation in length of practice; some participants were in the process of completing their final training and others had more than twenty-five years experience.

### Data collection and analysis

In total, fifteen focus groups and sixty interviews were conducted. Transcripts were stored and managed using the NVivo 7 software program [25]. Content and thematic analyses were undertaken. We employed a modified version of the framework approach [26], whereby a pre-defined set of guiding objectives was used alongside an inductive process to data collection and analysis – accommodating and exploring new themes as they emerged in the field.

Firstly, the transcripts were read several times and codes developed to cluster participant’s responses to topics within the interview schedule. Next, a number of themes were identified that captured patterns in participant responses, which were refined by members of the research team. Transcripts were coded according to the agreed themes and a strategy of mapping and interpretation was undertaken whereby data was examined in the context of the research questions and associations between themes identified, as were negative cases, meaning data that contradicted central themes [27].

## Results

The results are reported under three main headings that reflect the research questions, with each heading being broken down into various themes. The three main headings are: patterns of service usage, issues faced by carers in use of services, and improving service provision.

### Patterns of service usage

The general pattern of usage that emerged from the data was that family carers did not often use services early in the course of their loved ones’ dementia. However, they were prepared to use many of the services when their caring role became extremely stressful, when they became familiar with what was available and when they were able to feel comfortable about accepting this form of help. Family carers used a range of services, including home-based personal care and domestic help, home-based and centre-based respite and residential care.

#### Concerns about residential care

While family carers expressed some reservations about accepting home-based and respite services, the strongest reservations were about residential care. A large part of the belief about residential care was a strongly held view within all language groups that families should be looking after the people with dementia.

"You know we Chinese don’t like to send old people to nursing homes. We are still able to look after them. My father stays with us. You know. I know some places, but you know we don’t like to send our older generation to nursing homes. (Chinese family carer)"

Apart from the reservations based on cultural beliefs, family carers and health professionals discussed other concerns they had with residential care. These included perceptions of residential care as failing to cater to the person as an individual and that some residential care staff were predominately focused upon residents’ basic needs, like feeding and washing, at the expense of a more wholistic approach to care. Some carers suggested staff would neglect residents, including limiting the amount of times the resident was taken to the toilet.

The majority of family carers implied that placing their relative in residential care was a last resort. This may occur if the carer became exhausted or unwell, the carer’s other relationships were suffering, the person with dementia had increasing care needs or else their behavior had become unsafe or unacceptable.

"I found with my mother-in-law, she got to the point that she nearly burnt my house down and I thought that was it, I had four young children at home and I just couldn’t take that risk anymore so I put her name down for a nursing home (Italian family carer)"

Family carers spoke about how they had chosen to place their relative in residential care but still spent significant periods of time at the facility. A few bilingual/bicultural workers also commented on this practice, seeing it as illustrative of families’ commitment to the person with dementia.

"when it comes to a point that he really couldn’t handle, and he has to put the person in a nursing home but he doesn’t mean that their role (.) terminates, they will go to the nursing home every day from eight to eight (Jacqui, Chinese BBW)"

While there were strong reservations about residential care, a number of family carers also expressed positive views. There was an acceptance that they could not cope once the person’s needs got past a certain point. Carers were concerned about one or more of the following issues: the safety of the person with dementia, aggression from the person or their own physical or mental health problems. There was also a realization that the person may get better care within a facility than what they could provide at home and that this could be provided in a safe and caring environment.

"I think it is better to send our relatives [with dementia] to nursing homes so that we can focus on our work while they are being cared for. At least they will be given the correct prescriptions, and I don’t need to worry about that. I think it is one of the best ways. (Chinese family carer)"

#### The roles of ethno-specific/multicultural and mainstream services

Family carers from all communities preferred to receive care from workers from the same language background when receiving home-based care. Bilingual workers from ethno-specific or multicultural agencies could provide a personal and tailored service because they had an understanding of the language and culture of the clients and their families.

"They were so isolated so they were waiting for the time that their worker could talk to them in their language, take them outside. So it created a very special bond (Alandra, Spanish speaking BBW)"

Within centre-based respite and residential care, the programs were able to provide culturally appropriate food and activities. The person with dementia was also interacting with others who shared their language, culture and history. Therefore, they did not feel abandoned and some had more social interaction than when living at home, particularly if it was a small family or there were times when they were alone in the house. One participant stated that other nursing home residents would reassure family carers about the care provided by the program.

"Another reason we are alright is the other residents: “No, she’s OK. We are from the same village. We speak about the language; I’m two years older than your mum. She’s OK”…, so it’s why the culturally appropriate care is a positive thing (Kelly, Chinese BBW)"

The main problems identified with ethno-specific services were the long waiting lists due to the high demand, the observation that staff were not always well trained in dementia and that some staff did not speak the language of the community that the service was catering to.

"if you put someone that just speaks the language without having the training, then it’s a very, very narrow type of service provision (Iman, Arabic BBW)"

Because of the limited number of ethno-specific services, many people from CALD communities used mainstream services. Participants recounted a number of barriers that they faced in using mainstream services. The biggest concern expressed by participants was that mainstream dementia services did not often have bilingual staff. This caused significant problems as the person with dementia had either never spoken English well or else had lost their English because of the dementia. This made any communication with staff very limited and frustrating.

"When the people get older or demented they just leave them to a mainstream dementia day care and nobody can talk to that elderly, they just sit there and wait for the time to pass and they go home. It is very sad because everybody talk in different languages. (Catherine, Chinese BBW)"

The lack of culturally familiar food was seen as a barrier to using mainstream services. Italian bilingual/bicultural workers and family carers suggested that community members found food provided by ‘Meals on Wheels’ and food in residential care to be unpalatable.

"I bring her some Italian food, because what she eat there is smashed potatoes… [sic] I said to them “Why you give her this stuff?” (Italian family carer)"

Italian and Arabic carers had difficulties with aspects of community services. Family carers, bilingual/bicultural workers and geriatricians all described how Italians found the standard of cleaning provided by home care to be unacceptable. Arabic communities struggled with having an outsider come into the home and some Arabic family carers stated that personal care, even by someone of the same gender, was unacceptable. Arabic community members mentioned religion as a barrier to services, particularly for Muslims. Both care provision by a person of a different gender and sitting in a group (like a day-care facility) or sleeping in a room with someone of the opposite gender were seen as incompatible with their faith.

### Issues faced by carers in use of services

#### Unfamiliarity with the concept of formal services

Most participants suggested that people from their communities had little understanding of community-based and residential services for dementia. The first reason for this was because dementia was not recognized as a significant issue requiring health care; and the second reason was that community/welfare services do not exist or are very different to the form of service available in their country of origin.

"in China and other Asian countries, we don’t have that kind of aged care systems. If we are wealthy we can employ someone privately to come to help us, but in here things are different. It’s a lot better in, you know, a [this] place, so they have to change their concept from China, Asian country back home to here (Rachel, Chinese BBW)"

Carers from CALD backgrounds may perceive negative aspects of receiving services that would not be obvious to non-CALD service providers. For example a Chinese-speaking geriatrician explained that in China receiving community services only occurred if you were quite poor and did not have a family to support you, which meant that people in Australia may be reluctant to use them because it suggested they were poor and without family support.

Bilingual/bicultural workers noted that, due to a lack of familiarity with services, CALD communities do not readily seek assistance with home help or personal care of a family member.

"they don’t want to actually accept it because they don’t understand it. I mean, would you accept that you don’t understand? It’s the same thing, I mean, once our put yourself in someone else’s shoes, you realize exactly where they are coming from (Isi, Italian BBW)"

A number of bilingual/bicultural workers and GPs (except for Italian participants) explained that the idea of formal services was a new one for families and as a result they spent a lot of time discussing aged care services.

"So this is the way we take things, calmly, decently, bit by bit, step by step, explaining to them that we have respite, we have community services we are here to help (Hassan, Arabic BGP)"

#### Navigating complex service systems

Even if carers became familiar with the idea of services and were prepared to consider using them, it was often difficult to understand how the care system worked and how they could find services that met their needs. It was often difficult to get basic information about services. Some services had long waiting lists and there was considerable inequity in the availability of services across geographical areas.

"But it’s important to point out that it’s so disjointed and that if you don’t do your own research it’s a really hard system to navigate. I think it’s worth it to point out and if you end up getting someone who doesn’t give you that information years go by and you didn’t know that existed (Italian family carer)"

Carers really appreciated bilingual/bicultural workers, GPs and other staff who worked with them to follow up referrals and ensure that families were matched up with services that suited their needs. These bilingual professionals suggested that even when they had helped to clarify understandings and families had accepted some services, the families still required time to feel comfortable using these services.

#### Cultural traditions affecting the use of services

There were several features of people’s cultural background that influenced their perception and use of services. The first of these is the concept of ‘filial piety’ that adult children should obey and care for their elderly parents [28]. In our study, this belief was particularly identified by family carers and bilingual/bicultural workers within the Chinese and Arabic communities.

"we have another cultural aspect where in Arabic culture where the family have an obligation to look after their parents, as their parents brought them up (Iman, Arabic BBW)"

Another feature was what Spanish speakers term ‘familismo’, meaning that a family’s needs, or the needs of young or older family members take priority over individual needs [29]. This appeared particularly in the discussions of family carers and bilingual/bicultural workers from the Italian and Spanish speaking communities. A third feature, mentioned by participants of Italian and Spanish speaking backgrounds, was discussion of feelings of guilt or pressure to provide care at home. These participants explained that these feelings were both internalized and informed by cultural traditions.

"yeah but also there is a cultural connotation because since little we know we are to look after our parent, I mean I am married to an Australian man and when my Mum started to deteriorate for them it was easy, natural to think of a nursing home for me it was devastating, for them there was not guilt (Spanish family carer)"

A number of bilingual/bicultural workers and GPs from all communities, although Arabic-speakers to a more limited extent, suggested that Western influences had shifted some community members’ perceptions of residential care, with some more willing to consider nursing homes. Geriatricians attributed the differences amongst communities to acculturation as well as individual differences. Several geriatricians suggested the length of time that both individual families and communities had been in Australia impacted on their willingness to use services.

### Improving service provision

#### Community education about dementia and related services

Participants identified a strong need for more information about dementia and dementia-related services. This needed to include information in their own language as well as being more readily available within the community.

"I think the government can work more on the distribution of information so that every one of us will know about services that the government provides. At the moment, we have no idea what is available to us (Chinese family carer)"

#### Supporting families on the pathway to accepting services

Bilingual/bicultural workers, GPs and staff in dementia-related services often have to work with individuals or families for a period of time before they finally accept the need for and begin receiving services. The professional staff typically need to develop a trusting relationship and to be sensitive to the circumstances and needs of the person with dementia and their family carers. Bilingual/bicultural workers and GPs explained that they had spoken to family carers about their experiences of caring for the person with dementia. These health professionals identified three core features of such conversations: reassuring families they should not feel guilty, acknowledging the seriousness of the person with dementia’s behavior and validating families’ concerns about not coping.

"If we don’t explain to them, they don’t know. So how are they going to go and grab a brochure if they don’t know that there’s anything…old people because they are not used to services, sometimes they are very reluctant at the beginning to take the service. Then they love it and they want more hours and more hours (Christina, Spanish BBW)"

When discussing residential care with families, the health professionals suggested they were mindful of families’ attitudes to care and made a point of making a slow introduction to such services. As outlined by the respondents, there were two main features of these discussions: that dementia is a progressive condition and that more care will gradually be required; and secondly, stressing the benefits of residential care for the person with dementia rather than the carer, as this was seen as likely to be more culturally acceptable.

"look, I’m not trying to do what’s in your best interests, but in the best interests of the patient, ah to consider” and sometimes you are successful, sometimes you’re not (Sami, Arabic BGP)"

#### Ensuring a mix of ethno-specific/multicultural and mainstream services

There was a clear preference from most family carers to use ethno-specific or multicultural services over mainstream services. The reasons cited for this were that they received a more personal level of care, could talk to staff and other clients in their own language, had access to food and activities that were familiar to them and felt more comfortable with people who understood their cultural background. While this service model is highly valued, it is not sustainable to provide all dementia care in this way. Some bilingual/bicultural workers who were employed in mainstream services suggested their workplaces had good models of service delivery and that mainstream services should work in partnership or broker with ethno-specific and multicultural services. These latter services often have staff who act as a bridge between CALD communities and mainstream aged care organizations.

"we also have another very important colleague who connects the ethno-specific service and the members of their community and their needs in other organizations and other public and private sectors such as hospitals, referrals to maintain the comfort of people in nursing homes and looking after dietary and cultural needs (Iman, Arabic BBW)"

#### Helping mainstream services to become more culturally sensitive

A number of suggestions, particularly from bilingual/bicultural workers and family carers, related to how mainstream services could be improved. It was seen as important for a service to be aware of the makeup of their local community and to employ staff likely to be able to speak the languages of regular users. When agencies provide a one-to-one service, for example in-home respite, efforts should be made to use staff who can speak the client’s language, even if this means using contract staff. All staff should receive training to increase their awareness of and sensitivity to clients’ cultural backgrounds. Services need to be aware that families may have different support structures and make allowance for this, for example having a number of carers involved from within an extended family rather than just a single carer. When planning meals and program activities, services need to provide a range of options that reflect the cultural diversity of service users.

A few family carers suggested that mainstream services could meet their relative’s needs by calling on them when assistance was required

"they’ll ring me and say “look, your Mum won’t have a shower” and it’s been three days, we don’t know what to do, I go down there and shower her, I don’t care as long as they tell me (Italian family carer)"

Finally, it was suggested that monolingual workers could provide better care by learning some essential words in the client’s language, taking the time to read the person with dementia’s facial expressions or practicing non-verbal communication.

#### Strengthening the role of GPs as a point of referral to services

Most people with dementia are seen by their GP sooner or later in the course of their condition. While many carers described the help they received from their GP in positive terms, there were also many who perceived GPs as not adequately acknowledging the dementia, as unaware of support services in the community, or else not able to invest the time to explore the needs of patients and families and make appropriate referrals.

"I don’t think that the doctors really acknowledge it enough…explain to them enough, talk to them enough about it either so, the local doctors will often say to the family ‘Well you know, yeah your parents are getting old or whatever, yeah it’s to be expected. But they would be better off giving them more information about it so yeah, there’s definitely not enough, enough understanding about it (Salvagia, Italian BBW)"

While acknowledging the frequent time pressures in GP practices, it was suggested that GPs could liaise more with multicultural workers and CALD community organizations in their area, provide more information about dementia and services to patients and families and allow the time to do more comprehensive assessments of people with dementia.

## Discussion

The findings from this study to some extent reinforce those of other studies [5-13,15,18] as well as adding new insights and perspectives on the issues. One issue that emerged from the focus groups was that, while there was considerable common ground amongst participants, there was also variation in their attitudes, beliefs and behaviors. Apart from one’s cultural background, people are influenced by other factors such as education, family background, class, economic circumstances and migration experiences [30-32]. The family carers within our focus groups had a range of backgrounds, experiences and attitudes, which made this heterogeneity obvious. The implication of this is that service providers must not make assumptions about a person’s beliefs and behaviors based only on their cultural or language background.

A second issue that emerged from the fieldwork was the need to not assume all CALD communities have the same issues and needs as each other. The focus of this study was not to compare the beliefs and behaviors of the four communities, so we have not been able to report on such differences in detail. While we found there was considerable overlap in the views held with the four language communities, there were also significant differences. These differences will be influenced by a number of issues, including: the core cultural and religious beliefs of each community; the length of time the community has been in Australia; the degree of similarity between their own culture and mainstream Australian culture; and the amount and type of ethno-specific resources available within each community.

While there is good reason to support both ethno-specific and mainstream dementia programs, neither will provide all the answers alone. The number and geographical spread of people from CALD backgrounds requiring dementia-related services will only increase. It is particularly difficult to provide specific services for smaller CALD communities. While continuing to promote ethno-specific/multicultural services, it is also vital to ensure that mainstream services can also provide the best culturally and linguistically appropriate care possible. We support the greater adoption of a partnership model in dementia care, as proposed by Radermacher, Feldman, & Browning [19], where ethno-specific, multicultural and mainstream services work together and build models of good practice. Funded GP or GP practice nurse involvement in these partnerships would promote a more holistic approach to CALD dementia care in general practice.

Other findings which are similar to previous studies [5-13,15,18] include: the different understandings of dementia that may be held in CALD communities; carers’ lack of familiarity with and knowledge of dementia services; cultural beliefs about care within the family that means people often do not seek services until the needs are critical; carers’ difficulty accepting residential care; the important linking role played by bilingual health professionals; and preference for care within ethno-specific services, or at least mainstream services that were making a genuine attempt to cater to the cultural background of people in their care.

Apart from reflecting previous research, our study makes a new contribution to the literature through fresh insights to the experience of CALD community members and their use of dementia-related services. It is clear that CALD families struggle with care-giving and whilst some have a preference to keep the person with dementia in their home, there is a willingness to utilize community services that will enable them to do so. Family carers will use residential care as a last resort but will often feel guilty and reluctant to do this, struggling with both the personal, emotional and financial aspects as well as the reactions of others. CALD communities would benefit from education and support about residential services (what they are intended to do and reasons for placement) and individuals may need some supportive counselling as they are making a decision that may be seen as opposing their family or cultural values.

Our study has highlighted the importance of taking time to engage families, explain the nature of services and help them work through concerns they may have about using these. This is an important role – typically undertaken by a bilingual/bicultural worker, GP or dementia care staff – that needs to be more clearly acknowledged and supported. In terms of GP involvement, specific Medicare funding for these kinds of comprehensive assessments in people with cognitive impairment, including funding for discussion with the carer independently (which are currently not funded at all) might promote this approach.

An interesting outcome of our study is that improving dementia-related services to CALD communities requires input and commitment from a range of stakeholders. CALD community organizations can provide greater information about dementia through their existing information channels. They can also initiate and sponsor programs to provide dementia-related services to their community, as well as working with mainstream organizations on joint projects.

Government funding bodies need to allocate resources equitably to create a balance of ethno- specific and mainstream services, while service providers have a responsibility to provide programs that reflect the makeup of their local populations as well as facilitating access to these programs. This may be through a combination of sponsoring ethno-specific services and ensuring that their mainstream services are relevant and accessible for people from CALD backgrounds.

The study has several limitations. Information about the types of services used by carers is of a general nature rather than being detailed and quantifiable. The design did not include carers from the non-CALD mainstream community so it has not been possible to discuss issues for CALD communities in comparison to the mainstream community. This study was of an exploratory nature so provides useful insights rather than findings that can be generalized to all CALD communities. Further research needs to include other communities and explore aspects of service provision that are relevant for these communities as well as exploring how issues within CALD communities relate to issues within non-CALD communities.

## Conclusion

The research examined different stakeholders’ experiences and perceptions of service provision for dementia in four CALD communities. It is evident that formal services are very important for CALD communities and families will utilize those that they see as culturally and linguistically appropriate and able to meet their needs. Rather than rejecting services, most CALD communities have limited knowledge about the Australian aged care and dementia service systems, and ongoing education in this area is vital. In terms of the type of services used, people from CALD communities are most comfortable using services that will allow them to keep the person with dementia at home. However, some families will, if the approach and delivery of information is appropriate, consider residential services. All stakeholders suggested ethno-specific services were important in meeting community needs, but also that these needs can be met when mainstream services employ or broker bilingual/bicultural staff and provide culturally relevant resources and activities. The challenges in providing dementia-related services to CALD communities will best be met with ongoing commitment from a range of stakeholders. These include CALD community organizations, mainstream health, community and dementia-specific services as well as government policy and funding agencies.

## Competing interests

The authors declare that they have no competing interests.

## Authors’ contributions

DB was involved in conception and design, data collection, analysis and interpretation, and drafting of manuscript. CS, JA and YS were all involved in conception and design, analysis and interpretation, and drafting of manuscript. HK, DP and JR were all involved in conception and design and reviewing draft papers. All authors read and approved the final manuscript.

## Pre-publication history

The pre-publication history for this paper can be accessed here:

http://www.biomedcentral.com/1472-6963/12/354/prepub
